# Common T-Cell-Receptor Motifs and Features in Patients with Cytomegalovirus (CMV)-Seronegative End-Stage Renal Disease Receiving a Peptide Vaccination against CMV

**DOI:** 10.3390/ijms23031029

**Published:** 2022-01-18

**Authors:** Lukas Bunse, Claudia Sommerer, Chin Leng Tan, Felix Korell, Anita Schmitt, Angela Hückelhoven-Krauss, Brigitte Neuber, Thomas Mertens, Michael Platten, Edward W. Green, Martin Zeier, Michael Schmitt

**Affiliations:** 1 DKTK CCU Neuroimmunology and Brain Tumor Immunology, German Cancer Research Center (DKFZ), 69120 Heidelberg, Germany; lukas.bunse@umm.de (L.B.); c.tan@dkfz-heidelberg.de (C.L.T.); michael.platten@umm.de (M.P.); e.green@dkfz-heidelberg.de (E.W.G.); 2 Department of Neurology, MCTN, Medical Faculty Mannheim, Heidelberg University, 69117 Heidelberg, Germany; 3 Division of Nephrology, University Hospital Heidelberg, Renal Clinic Heidelberg, 69120 Heidelberg, Germany; claudia_sommerer@web.de (C.S.); Martin.Zeier@med.uni-heidelberg.de (M.Z.); 4 Faculty of Biosciences, Heidelberg University, 69117 Heidelberg, Germany; 5 Department of Internal Medicine V, University Hospital Heidelberg, 69120 Heidelberg, Germany; Felix.Korell@med.uni-heidelberg.de (F.K.); Anita.Schmitt@med.uni-heidelberg.de (A.S.); Angela.Hueckelhoven-Krauss@med.uni-heidelberg.de (A.H.-K.); Brigitte.Neuber@med.uni-heidelberg.de (B.N.); 6 Institute of Virology, University of Ulm, 89081 Ulm, Germany; thomas.mertens@uni-ulm.de; 7 Immune Monitoring Unit, DKFZ and National Center for Tumor Diseases, 69120 Heidelberg, Germany

**Keywords:** CMV, end-stage renal disease, TCR motif, peptide vaccination, single-cell sequencing

## Abstract

After solid-organ transplantation, reactivation of the cytomegalovirus (CMV) is often observed in seronegative patients and associated with a high risk of disease and mortality. CMV-specific T cells can prevent CMV reactivation. In a phase 1 trial, CMV-seronegative patients with end-stage renal disease listed for kidney transplantation were subjected to CMV phosphoprotein 65 (CMVpp65) peptide vaccination and further investigated for T-cell responses. To this end, CMV-specific CD8^+^ T cells were characterized by bulk T-cell-receptor (TCR) repertoire sequencing and combined single-cell RNA and TCR sequencing. In patients mounting an immune response to the vaccine, a common SYE(N)E TCR motif known to bind CMVpp65 was detected. CMV-peptide-vaccination-responder patients had TCR features distinct from those of non-responders. In a non-responder patient, a monoclonal inflammatory T-cell response was detected upon CMV reactivation. The identification of vaccine-induced CMV-reactive TCRs motifs might facilitate the development of cellular therapies for patients wait-listed for kidney transplantation.

## 1. Introduction

Patients receiving a kidney transplantation are prone to cytomegalovirus (CMV) reactivation. The most vulnerable time period is early after transplantation when patients are under high immunosuppression [1], where up to half of these patients can develop symptomatic CMV disease. CMV reactivation and disease are associated with increased long-term morbidity, graft loss [2,3] and mortality [2,3]. Following transplantation, patients with the serostatus donor^+^ recipient^−^ (D^+^R^−^) are at highest risk, while D^+^R^+^ and D^−^R^+^ transplantations constitute an intermediate risk.

Classical prophylaxis or preemptive therapy using (val)ganciclovir or foscavir is effective, with nephron- and myelotoxicity limiting the use of these drugs [4].

In addition to drug treatment, vaccination offers an intriguing option in development for patients at risk [5]: several CMV vaccines are currently under investigation in phase I to phase III clinical trials, featuring attenuated viruses and truncated proteins as well as DNA vaccines [6,7,8,9,10,11]. The cellular immune response is essential for controlling CMV infection [12]. Patients might be protected once a detectable T-cell response against CMV has been reached. 

Recently, we published the results of a first phase I trial for a CMVpp65-derived vaccine in HLA-A*02-positive CMV-seronegative end-stage renal disease patients on the kidney transplant waiting list [13]. The study demonstrated that the HLA-A*02-restricted CMVpp65-peptide vaccine application was safe, was well tolerated and showed clinically encouraging results in these high-risk patients.

However, further investigation of both the affected patient cohort and of possible response factors is highly warranted. 

For this reason, we aimed at a deeper understanding of T-cell-receptor (TCR) repertoire dynamics and specificities and, in addition, report here on the characterization of CMV-specific CD8^+^ T cells by bulk TCR sequencing as well as combined single-cell RNA and TCR sequencing.

## 2. Results

### 2.1. Impact of CMV-Peptide Vaccination on the TCR Repertoire

**Figure 1:**
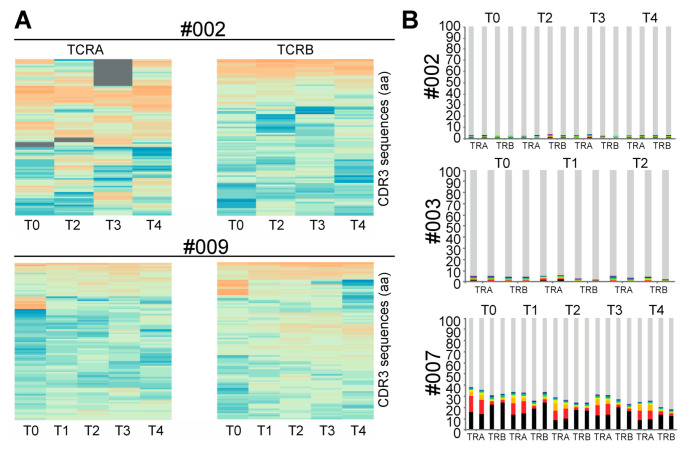
Deep TCRA and TCRB sequencing for patients’ PBMCs in CMV-seronegative end-stage renal disease. (**A**) Top 100 TCRA and TCRB sequences from responder (#009) and non-responder (#002) patients visualized at baseline (T0) and three post-vaccine timepoints (T2, T3 and T4). Two exemplary patients are shown. Gray, respective clonotypes were not found at time points illustrated. (**B**) Proportion of the top-ten clonotypes from responder (#003, #007) and non-responder (#002) patients visualized at baseline (T0) and two to four different post-vaccine timepoints (T1–4). Samples were sequenced in technical duplicates.

To understand the clonal evolution of T-cell clonotypes following repetitive CMVpp65 peptide vaccinations and to identify TCR sequences dominating peptide-induced peripheral T-cell responses, we performed deep repertoire TCRA and TCRB sequencing. In both responder and non-responder patients, repetitive vaccinations were not associated with consistent longitudinal alterations in the most-abundant clonotypes in the peripheral blood (Figure 1). One responder patient (#007) had a number of dominant clones in the pre-vaccination repertoire, suggesting a pre-existing immune response to an independent immune challenge, which decreased over time. In contrast, the top clonotypes in non-responder patients #001 and #002 were stable during repetitive vaccination. Taken together, in our study, the repetitive CMVpp65 peptide vaccination of patients with CMV-seronegative end-stage renal disease did not alter the frequency of the most-abundant TCR clonotypes in the peripheral blood.

**Figure 2:**
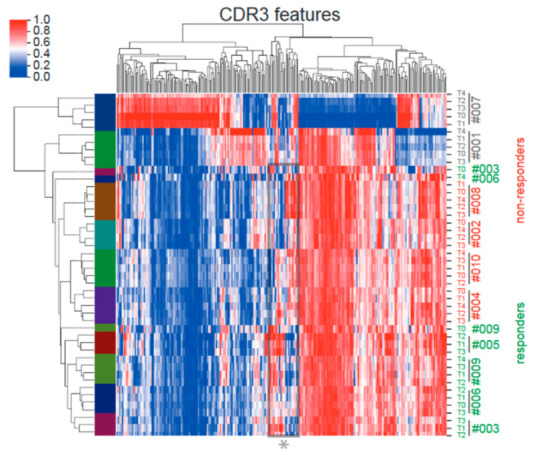
Unsupervised deep-learning-based clustering of patient repertoires using DeepTCR. Ten CMV-seronegative end-stage renal disease patients waiting for kidney transplantation were vaccinated four times biweekly. #001 and #007 represent patients with existing strong immune responses. Patients classified as responders (#003, #005, #006 and #009; green labels) and non-responders (#002, #004, #008 and #010; red labels) by CMV-tetramer binding formed distinct clades, with a number of CDR3 features (*) best correlating with response.

**Figure 3:**
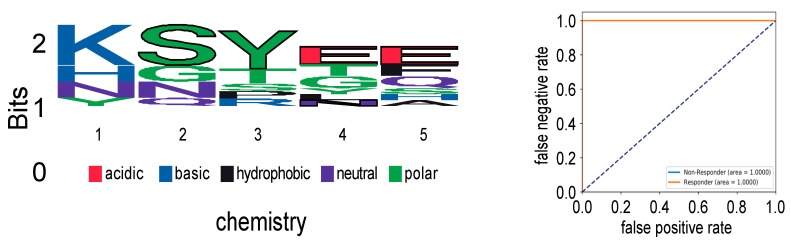
A “SYE(N)E” motif (**left**) harboring polar, acidic and neutral amino acids known to bind HLA-A*02-presented CMVpp65 was found only in responders but not in non-responder patients (**right**).

To assess the impact of CMVpp65 peptide vaccination on the TCR repertoire in more detail, we performed unsupervised deep-learning-based clustering of patient TCR repertoires using DeepTCR [14]. The featurization of TCRs enables the identification of antigen-specific TCR features in biologically noisy TCR repertoires with a high abundance of irrelevant T cells. Additionally, pre-vaccination TCR features can then be interrogated as predictors of response. Whereas patients #007 and #001 each had unique enrichments of CDR3 features throughout the TCR repertoire distinct from all the other patients receiving CMVpp65 peptide vaccination, the remaining eight patients globally showed similar CDR3 feature enrichment within their repertoires including the pre-vaccination time point samples (T0). However, the retrospective classification of response (#003, #005, #006 and #009) and non-response (#002, #004, #008 and #010) by CMV-tetramer staining enabled the identification of distinct response-associated CDR3 features (Figure 2). More specifically, a “SYE(N)E” TCR motif known to bind HLA-A*02-presented pp65 (vdjdb.cdr3.net (accessed on 15 August 2019) was found in responders, making it a predictor of response (Figure 3).

### 2.2. Combined Single RNA and TCR Sequencing in Follow-Up

**Figure 4:**
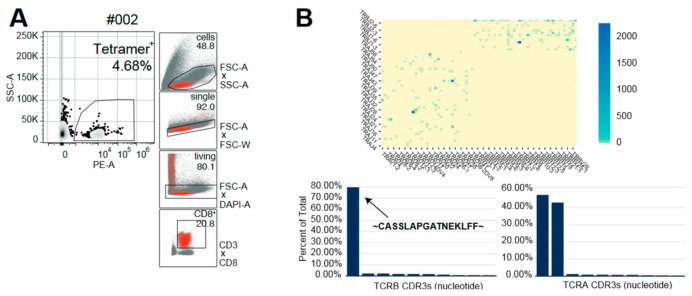
Single-cell TCR sequencing upon CMV reactivation in non-responder patient #002 five months after transplantation. Combined scTCR-seq and scRNA-seq for HLA-A2*-tetramer-sorted CMV-reactive circulating peripheral T cells after CMV reactivation. (**A**) Tetramer sorting gating strategy for CMV-reactive circulating peripheral T cells. (**B**) TCRA and TCRB gene usage heatmap (**left**) and clonotype percentages (**right**) of CMV-reactive T-cell clonotypes. Most-abundant TCR beta chain is illustrated as amino-acid sequence.

**Figure 5:**
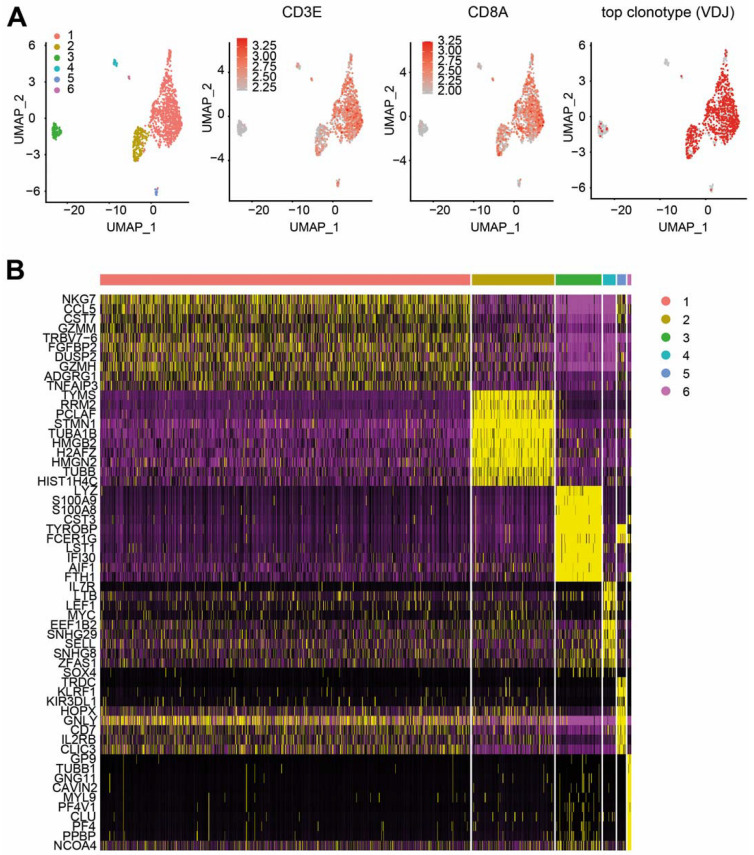
Combined scRNA-seq and scTCR-seq of patient #002-dominating CD8^+^ CMV-reactive T-cell clonotype. (**A**) Six differential T-cell clusters of patient #002-dominating CD8^+^ CMV-reactive T-cell clonotypes visualized by UMAP. Expression levels of CD3E and CD8A are shown. Co-visualization of the top TCR clonotype and its distribution within T-cell clusters visualized by UMAP. (**B**) Heat map of differentially expressed genes in clusters.

Five months after transplantation, non-responder patient #002 experienced CMV reactivation. To obtain a more detailed immunological understanding of this reactivation, we performed combined single-cell (sc) RNA (scRNA) and scTCR sequencing of CMV-reactive PBMC-derived T cells. Upon reactivation, the patient mounted a monoclonal (80%) CMV-specific T-cell response with a unique TCR beta chain pairing with two different alpha chains (Figure 4). Furthermore, the combined scRNA/scTCR-seq of the patient #002-dominating CD8^+^ CMV-specific clonotype revealed two predominant transcriptional states highlighted by a high degree of both effector function (NKG7^high^, CX3CR1^high^ and GZMB^high^) [15,16] and proliferative capacity (TYMS^high^ and MKI67^high^) (Figure 5, Supplementary Table S1). Using the software package ALICE [17], calculating the generation probability for CDR3s using OLGA [18], we further explored whether other signals of convergent CDR3 selection in patient #002 occurred during repetitive CMVpp65 and CMV reactivation. Interestingly, we found evidence of multiple convergently selected CDR3 sequences such as CDR3, CASSAGTGTYEQY, which was previously reported to bind the IE1 ‘KLGGALQAK’ epitope presented on HLA-A*03 (vdjdb.cdr3.net (accessed on 1 November 2021)).

**Figure 6:**
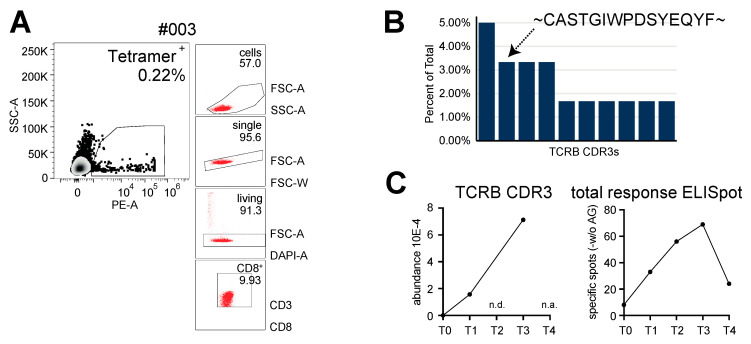
Combined scRNA-seq and scTCR-seq of CMV-reactive T cells from vaccine-responding patient #003. (**A**) Tetramer sorting gate for CMV-reactive circulating peripheral T cells. (**B**) Frequency of top-ten TCR clonotypes within the enriched CMV-reactive T-cell repertoire. Amino-acid sequence of the second-most-abundant clonotype is shown. (**C**) Clonotypic evolution of the second-most-abundant sc-sequencing-retrieved TCR for longitudinal TCRB deep-sequencing datasets (**left**) and longitudinal peripheral pp65-specific ELISpot responses (**right**). n.d., not detectable; n.a., not assessed.

In contrast, the scTCR-sequencing of tetramer-sorted CMV-reactive T cells from vaccine-responding patient #003 revealed that the second-most-abundant post-enrichment clonotype was found at all the available post-vaccine time points, and its abundance peaked, in congruence with the peripheral total T-cell response, at time point 3 (Figure 6). Of note, the most-abundant scTCR-sequencing clonotype from patient #003 constituting up to 5% of the tetramer-sorted CMV-reactive TCR repertoire was not found in the deep TCRB PBMC sequencing datasets.

## 3. Discussion

CMV reactivation after solid-organ transplantation constitutes a serious clinical problem [19]. It is known that cellular immunity through effector cytotoxic and helper T cells plays a critical role in controlling CMV replication after transplantation [20].

Within our phase 1 trial, ten patients received four subcutaneous vaccinations as per protocol. The vaccine was well tolerated, and 5/10 patients mounted a T-cell response with an emulsified CMVpp65 nonamer peptide [13]. Further investigation of both the affected patient cohort and of possible response factors is highly warranted. 

For this reason, we analyzed the T-cell response to CMV-specific peptide vaccination in exemplary patients in more detail for a deeper understanding of T-cell receptor (TCR) repertoire dynamics and specificities and the characterization of CMV-specific CD8^+^ T cells by bulk TCR sequencing as well as combined single-cell RNA and TCR sequencing.

Previous reports had demonstrated that immunodominant CMV antigens can induce highly diverse changes in T-cell-receptor (TCR) repertoires, which, nevertheless, contain convergently selected motifs and/or public clones indicative of patient response and HLA type [21,22,23]. In both responder and non-responder patients, repetitive vaccinations were not associated with longitudinal alterations in the most-abundant clonotypes in the peripheral blood (Figure 1). Therefore, we performed an unsupervised deep-learning-based clustering of TCR repertoires using DeepTCR [14]. Including pre-vaccination timepoints (T0), responders and non-responders formed distinct clades in TCR cluster analysis (Figure 2). Moreover, a “SYE(N)E” motif known to bind HLA-A*02-presented pp65 was found in responders as a predictor of response (Figure 3). In patient #002, at five months after transplantation, upon CMV reactivation, we sorted CMV-reactive circulating peripheral T cells (Figure 4A). A single clone constituted 80% of the repertoire of HLA-A*02 CMV-tetramer-positive T cells (Figure 4B). This clonotype was described in several studies as HLA-A*02 restricted and CMV reactive [23] but was not found in longitudinal TCRA and TCRB datasets for the same patient, suggesting an inability to mount a distinct clonotypic response as a mechanism of CMV reactivation. Whereas conclusions on disease severity from this single observation cannot be drawn, the generation of this monoclonal T-cell response might be a consequence of a profound CMV antigenemia that might represent a stronger immunological stimulus compared to the CMVpp65 peptide vaccine. However, we found evidence of multiple convergently selected CDR3 sequences such as CDR3, CASSAGTGTYEQY, which was previously reported to bind the IE1 ‘KLGGALQAK’ epitope presented on HLA-A*03 (vdjdb.cdr3.net). As patient #002 is also HLA-A*31 positive and HLA-A*03 and HLA-A*31 fall into the same HLA supertype family, it remains unknown if this observation is causally related to both the previously reported ineffective T-cell response (tetramer staining) but, at the same time, weak T-Track™ [24] assay results [13].

In general, transplantation-associated immunosuppression interferes with anti-CMV immune responses [20]. Specifically, it has been shown that T-cell-depleting agents increased the risk for CMV infection due to the direct depletion of functional CMV-specific T cells. Conversely, it has been reported that a release of proinflammatory cytokines is capable of activating latent CMV infections. Mycophenolic acid (MA) inhibits lymphocyte activation that facilitates CMV infection, especially in high doses [25]. MA treatment decreases both primary and secondary humoral immune responses [26]. In a previous study, MA reduced the seroresponse of kidney transplant recipients to pandemic H1N1 vaccination [27]. Importantly, while T-cell-depleting agents were prohibited in this phase 1 clinical study, the use of MA might have influenced the post-vaccination cellular immune responses. 

Vaccination strategies have been highly efficacious for several decades in controlling infectious diseases, highlighted more recently by the COVID-19 pandemic. Vaccines that include whole organisms or large proteins appear to have some adverse side effects attributable to the inclusion of an unnecessary antigenic load [28]. In principle, a high antigenic load might increase the probability of allergenic responses. Peptide vaccination, however, is an attractive alternative strategy that relies on the usage of short peptide fragments to engineer the induction of highly targeted immune responses. On the other hand, and in contrast to mRNA vaccines, peptide vaccines are often weakly immunogenic and require adjuvants. In the recent study, the CMVpp65 peptide was emulsified with incomplete Freund’s Adjuvant (Montanide^®^) and combined with a local application of the Toll-like receptor (TLR)-7 agonist imiquimod (Aldara^®^ 5% cream) [13]. Both adjuvants had been used successfully and safely in other studies [29]. It remains unknown if the use of other adjuvants would have resulted in differential outcomes such as in non-responder patient #002, who demonstrated, in a follow-up analysis, in principle, an ability to mount a meaningful monoclonal response against CMVpp65 solely upon CMV reactivation.

In summary, 50.0% of the vaccinated patients mounted a peripheral immune response during prophylactic CMV-specific peptide vaccination prior to kidney transplantation. Although only a small number of patients were enrolled in this phase 1 clinical trial, CMV-associated TCR motifs could be identified. Further follow-up studies with an increased patient number and multi-center assessment are necessary to confirm the clinical as well as exploratory translational results. Future vaccination strategies might also incorporate MHC class II antigens or other immunotherapeutic strategies such as mRNA vaccines or TCR-transgenic cellular therapies.

## 4. Materials and Methods

### 4.1. Underlying Clinical Study

Detailed information for both the CMVpp65 peptide vaccine and the investigated patients has been described earlier in the published clinical results of the phase I peptide vaccination trial [13].

In this phase I study, 10 CMV-seronegative end-stage renal disease patients waiting for kidney transplantation were vaccinated four times biweekly. All the enrolled patients were CMV IgM/IgG negative prior to the CMVpp65 peptide vaccination. At baseline, the participants showed neither pre-existing CMV-specific CD8^+^ T cells in tetramer-based flow cytometry nor significant (>10/200.000) interferon gamma (IFN-γ) spot-forming cells (SFC). 

In 5 of 10 patients (50.0%; #003, #005, #006, #007 and #009), any immune responses, as evidenced by the detection of an increase in IFN-γ production in the T-Track™ assay and/or an increase in CMV-specific CD8^+^ T cells, were observed within the core study of 56 days lasting until 14 days after the last vaccination [13]. Patients #001, #002, #004, #008 and #010 were non-responder patients. Four patients developed CMV-specific effector T cells, and one patient developed significant immediate-early (IE)-1- and pp65-specific spot-forming cells in the IFN-γ ELISpot assay only (#007).

Details on the manufacturing process and administration of the vaccine as well as all the clinical results and side effects are provided in the previous publication [13].

### 4.2. Tetramer Staining for CMV-Specific CD8+ T Cells

The frequency of CMV-specific CD8^+^ T cells was determined as described earlier [30]. The acquisition was performed on an LSRII™ device (BD Biosciences, San Diego, CA, USA), and the analysis was performed using the BD FACSDiva™ software (BD Bioscience, San Diego, CA, USA).

### 4.3. Bulk T-Cell-Receptor Repertoire Sequencing and Analysis

A total of 5 × 10^6^ viable peripheral blood mononuclear cells (PBMCs) per time point for each patient were frozen in DMSO/freezing medium. Following thawing, RNA extraction was performed and the RNA was concentrated using a SpeedVac™ (Thermo Scientific^TM^, Waltham, MA, USA). Where sufficient RNA was available, duplicate random amplification of cDNA ends (RACE) reactions targeting the alpha- and beta-chains of the TCR (TCRA and TCRB) were set up using 250 ng of RNA as the template. Preliminary repertoire analysis was conducted with VDJ tools [31], and the CMV specificity was determined with reference to published CMV-specific TCRs listed in VDJdb (June 2017) [31]. The TCR repertoires were further analyzed using the publicly available tools ALICE/OLGA [17,18] and DeepTCR [14].

### 4.4. Single-Cell RNA and T-Cell-Receptor Library Construction and Sequencing

For one responder (patient #003) and one non-responder (patient #008), single-cell RNA-seq and single-cell TCR-seq libraries were prepared using the Single Cell Immune Profiling Solution Kit (10× Genomics, Pleasanton, CA, USA), according to the manufacturer’s instructions. For the gene-expression library construction, amplified cDNA was fragmented and end-repaired, double-sided size-selected with SPRIselect beads (Beckman Coulter), PCR-amplified with sample-indexing primers (10× Genomics, Pleasanton, CA, USA) and double-sided size-selected with SPRIselect beads (Beckman Coulter, Brea, CA, USA). For TCR library construction, TCR transcripts were enriched from 2 μL of amplified cDNA by PCR. Following TCR enrichment, the PCR product was fragmented and end-repaired, size-selected with SPRIselect beads (Beckman Coulter, Brea, CA, USA), PCR-amplified with sample-indexing primers and size-selected with SPRIselect beads (Beckman Coulter, Brea, CA, USA). The single-cell RNA libraries were sequenced on an Illumina HiSeq 4000™ (San Diego, CA, USA). The single-cell TCR libraries were sequenced on an Illumina NextSeq 550™ paired-end 150 mid-output flow cell to a minimum sequencing depth of 5000 reads per cell.

### 4.5. Single-Cell Data Analysis 

The sequencing data were aligned using cellranger (v6.1.2) (10× Genomics, Pleasanton, CA, USA). The count matrix was then imported into R for further downstream analysis. The data were normalized and transformed using the Seurat package sctransform and subsequently clustered via the Louvain algorithm using Seurat. A UMAP and heatmap were also plotted using Seurat (v.4.0.4).

### 4.6. IFN-γ ELISpot

Commercially available IFN-γ ELISpot T-Track^®^ CMV (Lophius Biosciences GmbH, Regensburg, Germany) assays were used for the assessment of CMVpp65 antigen-specific IFN-γ release as previously reported [13].

### 4.7. Statistical Analyses 

The exploratory results are presented in a descriptive manner, with numbers and percentages.

## Data Availability

The data presented in this study are available on request from the corresponding author. The data are not publicly available due to national law.

## Supplementary Materials

The following are available online at https://www.mdpi.com/article/10.3390/ijms23031029/s1.
